# Compact Wireless Microscope for *In-Situ* Time Course Study of Large Scale Cell Dynamics within an Incubator

**DOI:** 10.1038/srep18483

**Published:** 2015-12-18

**Authors:** Di Jin, Dennis Wong, Junxiang Li, Zhang Luo, Yiran Guo, Bifeng Liu, Qiong Wu, Chih-Ming Ho, Peng Fei

**Affiliations:** 1 School of Optical and Electronic Information, Huazhong University of Science and Technology, Wuhan, 430074, China; 2 Department of Precision Instrument, Tsinghua University, Beijing, 100084, China; 3 Mechanical and Aerospace Engineering Department, University of California, Los Angeles, Los Angeles, 90095, U.S.A; 4 Department of Bioengineering, University of California, Los Angeles, Los Angeles, 90095, U.S.A; 5 School of Life Sciences, Tsinghua University, Beijing, 100084, China; 6 School of Mechanical Science and Engineering, Huazhong University of Science and Technology, Wuhan, 430074, China; 7 College of Life Science and Technology, Huazhong University of Science and Technology, Wuhan, 430074, China

## Abstract

Imaging of live cells in a region of interest is essential to life science research. Unlike the traditional way that mounts CO2 incubator onto a bulky microscope for observation, here we propose a wireless microscope (termed w-SCOPE) that is based on the “microscope-in-incubator” concept and can be easily housed into a standard CO2 incubator for prolonged on-site observation of the cells. The w-SCOPE is capable of tunable magnification, remote control and wireless image transmission. At the same time, it is compact, measuring only ~10 cm in each dimension, and cost-effective. With the enhancement of compressive sensing computation, the acquired images can achieve a wide field of view (FOV) of ~113 mm^2^ as well as a cellular resolution of ~3 μm, which enables various forms of follow-up image-based cell analysis. We performed 12 hours time-lapse study on paclitaxel-treated MCF-7 and HEK293T cell lines using w-SCOPE. The analytic results, such as the calculated viability and therapeutic window, from our device were validated by standard cell detection assays and imaging-based cytometer. In addition to those end-point detection methods, w-SCOPE further uncovered the time course of the cell’s response to the drug treatment over the whole period of drug exposure.

Light microscopy is a widely used technique that brings insight into modern life science research by enabling visualization of microscopic phenomena. Numerous light microscopy techniques based on different principles have been invented in the past century^1^,^2^,^3^,^4^,^5^,^6^. In spite of the various modalities, microscopes in the common sense generally involve fairly complicated settings with large form factors and high upkeep. Therefore, for a long time, access to microscopes, especially fluorescent microscopes, has been limited to highly specialized sites, such as hospitals and research laboratories. Recently, several types of portable, cost-effective light microscopes have emerged^7^,^8^,^9^,^10^,^11^,^12^,^13^. Imaging with these portable microscopes is accomplished by using small optics and electronics^7^,^8^,^10^,^11^. In some modalities^9^,^12^,^13^, even the lens elements, generally the most essential components for imaging, are eliminated to drastically reduce the size of the device and to circumvent the need to find a proper balance between field-of-view and resolution^14^. To create an image with both high resolution and large FOV, a series of post-processing strategies, such as pixel super-resolution^12^,^15^, in-line digital holography reconstruction^15^,^16^ and compressive sensing^8^,^9^, are used to compensate for the unsatisfactory quality captured by the limited optical power. These compact and lightweight microscope devices for bright-field and fluorescent imaging are desirable for use in resource-limited environments^17^.

Most of the aforementioned compact microscope devices are optimized for stained dead cell analysis. These devices are exempt from the requirement of a dedicated environment with stable humility, temperature and CO_2_ concentration, which is necessary for long-term live cell observation. However, observing changes in live cells over a period of time, known as time-lapse or longitudinal microscopy, is essential to a variety of cell biology research areas. Examples of its uses include aiding in drug screening^18^, visualizing cell apoptotic processes^19^, analyzing cell division phenotypes^20^ and investigating gene function by RNA interference^21^. Currently, the dominating method to create a stable and suitable environment for cellular growth while concurrently observing the cells is to build a customized incubator on an existing microscope due to the infeasibility of bringing the bulky microscope into a CO_2_ incubator. Aside from the cumbersome form factor, the conventional incubator-on-microscope modality requires considerable expense due to the necessity of the special incubator. Meanwhile, time-lapse imaging of cell culture has an intrinsic need for wide FOV, to track a larger population of cells for better statistical analysis over extended periods of time. In contrast, the conventional microscopes frequently used for housing the incubator and accommodating the cell culture typically has a minimum magnifying power of two, which causes a limited FOV no larger than 40 mm^2^ in the acquired digital images. Image stitching techniques are usually employed in this case, to stitch multiple small frames into a single big one, to achieve sufficiently large FOV. For this method, any failed image necessitates repetition of the entire acquisition, requiring 100% reliability for each frame captured during the observation period^22^. Moreover, the system needs to be equipped with additional high precision motorized parts^23^, adding to the complexity of the system.

Recently, several compact, lens-based and lens-free imaging devices characterized by low cost and modestly large field-of-view have been reported for dynamic observation of living cells^23^,^24^,^25^,^26^,^27^,^28^,^29^,^30^,^31^. In lens-based modalities, the mini-microscope is portable and allows easy integration with a wide variety of pre-existing platforms, such as petri dishes, cell culture plates, and microfluidic bioreactors, for chronologically monitoring the cell dynamics^30^,^31^. In lens-free modalities, to harvest sufficient resolution in the recorded raw images^24^,^28^, microfluidic chambers were specially designed to culture the cells and more importantly, place them close to the image sensor surface. As a result, the FOV achieved in lens-free setting is essentially fixed and can be as large as the active area of the sensor. For further improving the native resolution limited by the pixel size of the image sensor, multiple shift-correlated images of the cells could also be created in lens-free modalities, by precisely scanning the illumination source^25^,^27^,^29^ or taking advantage of the inherent motion of the microorganisms^26^. In both lens-based and lens-free modalities, the acquired raw images need to be transmitted from sensor to computer via data cable. For lens-free imaging, a pixel super-resolution processing is additionally required to compose a final resolution-enhanced image from multiple low resolution frames.

Here, we propose a portable microscopic imaging modality that allows novel remote control, wireless data acquisition and large scale observation on the living cells with compliance to standard cell cultivation environment. This wireless microscope, termed w-SCOPE, has a palm size format integrated with cost-effective elements consisting mainly of consumer electronics and miniature optics. Its design is based on the infinite distance imaging principle and simply contains a pair of lens for imaging the micro-samples with tunable magnifying power. The w-SCOPE accommodates commonly used petri dishes for cell culture. Its compact design enables easy integration into most commercial CO_2_ incubators and convenient *in situ* living cell observation. The original images captured from the w-SCOPE have a wide field of view to cover large scale cells. At the same time, the employment of image post-processing can further compensate for the limited optical power from frugal optics and enhance the resolution of the image results significantly. The unique Wi-Fi capabilities of w-SCOPE allows for on-air image transmission from the closed incubator to an external computer or smartphone. In contrast to the standard “incubator-on-microscope” concept, the w-SCOPE provides a simple and robust way to implement “microscope-in-incubator” imaging. With the w-SCOPE, we can take a periodic series of living cell images at designated time intervals and conduct a time course analysis to discover a variety of cell dynamics, such as cellular apoptosis, cell motility and cell response to drugs. As a demonstration, we have conducted a sustained 12 hours imaging on living cancer cells to study their dynamic response under anti-cancer drug treatment.

## Results

### System Setup

**Figure 1:**
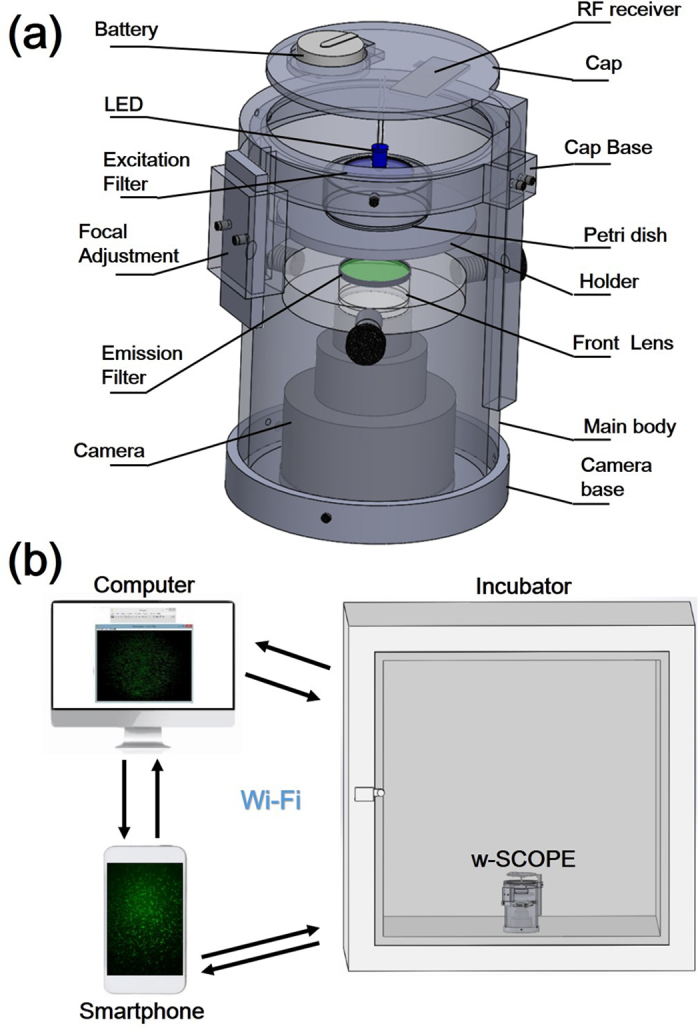
Schematic of the w-SCOPE device and its working principle. (**a**) Fluorescent imaging configuration of w-SCOPE. All the functional parts are labelled with full names. The key components include an LED as an illuminator, a set of filters for fluorescence collection, an achromatic lens doublet for microscopic imaging, a digital camera for data acquisition and transmission, and a 3D printed base for housing all the elements. (**b**) The working status of the w-Scope. The w-SCOPE is placed inside a CO2 incubator where a humidified 5% CO_2_ and 37 °C environment is maintained for cell culturing. A cell phone is used to wirelessly control the imaging process and record the sequential image data. The acquired images are stored in the cell phone memory in jpg format (typically 3–5 MB for an 18.2 mega pixel image), and can be either immediately viewed on the cell phone or transferred to a computer for further digital processing.

The detailed schematic of the w-SCOPE configured for fluorescent microscopy is shown in Fig. 1(a). The optical path is based on the principle of infinite distance imaging and the bracing structure of the device is created by rapid 3D printing. An achromatic doublet lens (Thorlabs) with a focal length of 30 mm and a diameter of 25.4 mm is placed in front of a QX10 camera (Sony) containing an 18.20 mega pixel CMOS sensor and a 10X zoomable lenses group. A petri dish that houses the cell sample is mounted onto the holder, and placed on the focal plane of the front achromatic lens. For focal adjustment, the vertical position of the petri dish holder can be finely adjusted along a specifically designed rail to reach the focal plane of the front lens, at which the camera can find the perfect focus on the cells in the center of the field. Then the petri dish is fixed at that position by the soft tip screw as shown in Fig. 1(a) during the whole subsequent imaging process. The final working distance is measured as ~25 mm from the bottom of the petri dish to the top of the front lens. The pair of the achromatic lens and camera’s built-in lens then images the cells onto the camera sensor plane with a tunable magnification from ~0.15 to ~1.5. A light-emitting diode (LED) powered by a coin battery is used as the illumination source. In the w-SCOPE’s bright field imaging configuration, white cold light emitted from the white LED travels a sufficient distance to form uniform and approximately perpendicular illumination, whereas in the fluorescent imaging configuration, a filter is attached to the colored LED for purer excitation light and another filter is placed over the front lens to wipe out excitation background. Simply by switching between different combinations of LEDs and filters, our device can flexibly achieve both bright-field imaging and fluorescent imaging. The full dimensions of the w-SCOPE are ~10 × 10 × 14 cm^3^. Though this size is compact enough for most commercial incubators, it can be further reduced in future optimizations.

To provide an adequate cell culture environment, during working, the w-SCOPE is placed inside a humidified 5% CO_2_ incubator at 37 °C as illustrated in Fig. 1(b). Before the start of observation, the w-SCOPE is pre-incubated for a few minutes inside, till it reaches the same condition with the incubator’s atmosphere. Then we wipe off the moisture accumulated on the optics and the cap of the petri dish, making them back to clear status. This step is performed once beforehand for removing the hazardous obscure by the condensation of humid air and needn’t to be repeated because the system will stay inside the incubator during the whole period of experiment. Although the w-SCOPE is completely sealed inside the incubator, wireless connectivity enables remote control, seamless data acquisition and image transmission to a smartphone outside the incubator. The entire cell monitoring process continuing up to days can be conducted wirelessly and non-invasively. According to the varying cell conditions, the device is able to switch between bright-field and fluorescent imaging with tunable exposure, tunable ISO, and tunable magnification. A maximum FOV of 188 mm^2^ can be achieved

### Imaging Capability Characterization

**Figure 2 f2:**
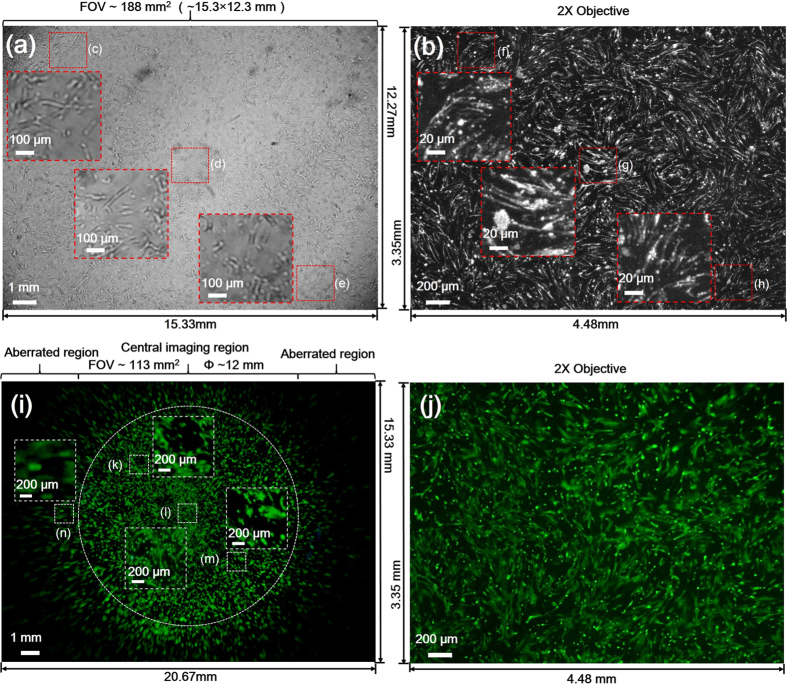
Wide FOV imaging of IMR-90 cells using w-SCOPE. (**a,b**) show the bright-field images taken by w-SCOPE with a 0.3X setting and conventional microscope using a 2X/0.06 objective respectively. The frame size of w-SCOPE image is ~15.33 × 12.27 mm, which is in comparison to ~4.48 × 3.35 mm by conventional microscope. Nearly the entire 188 mm^2^ FOV of w-SCOPE is effective under bright-field configuration. The inserted (c–e), and (f–h) display the selected areas of typical cells in (**a,b**), respectively. In contrast to (**a,b**), (**i,j**) correspondingly show the fluorescence images of IMR-90 cells labeled by CFDA SE cell tracer. As shown in (**i**), the edges of the image, which lie outside of the drawn central region, exhibit readily apparent aberrations and therefore are not counted into the effective FOV. The central aberration-free area of w-SCOPE image is ~113 mm^2^, which is smaller than bright field images, but still large enough for a variety of applications. It should be noted that for less demanding applications, the aberrant regions, such as inserted (n), could still be useful despite their lower image quality. (**j**) shows the same cells that are imaged by an inverted fluorescence microscope using a lowest power 2×/0.06 objective.

To characterize the FOV of the w-SCOPE, we imaged CFDA SE (live cell tracer, Life Technology) labelled IMR90 human fibroblast cells under both bright field and fluorescence configurations, with a magnification ~0.3X. The results are shown in Fig. 2. In the bright field trials, the captured image (Fig. 2a) displayed good quality with little aberration (Fig. 2c–e) over a large effective FOV of ~188 mm^2^. However, in the fluorescent image (Fig. 2i), due to the existence of weak emission area caused by the limited filtration aperture of excitation, more observable quality degradation occurred at the edges of the image (e.g., Fig. 2n), confining the effective FOV to the central imaging region of approximately 113 mm^2^. The aberration issue primarily caused by the insufficient size of the front lens doublet can be further mitigated through increasing the apertures. At the same time, although the region of the image with relatively small amounts of image distortion (Fig. 2n) is not as sharp as the center areas (Fig. 2k–m), it can still be useful in applications such as water quality monitoring^8^,^13^ where cell counting can be done despite the blurry cell outlines. The images captured through an inverted microscope (Nikon Eclipse TE-2000) using a low magnification 2X/0.06 objective and a middle frame CCD camera (Coolsnap HQ) are shown in Fig. 2b,j, respectively, as controls. The resulting FOV in bright-field and fluorescence images are both around 15 mm^2^ and can be further increased up to ~45 mm^2^ by using large format cameras.

### Resolution Enhancement Using Compressive Sensing

**Figure 3 f3:**
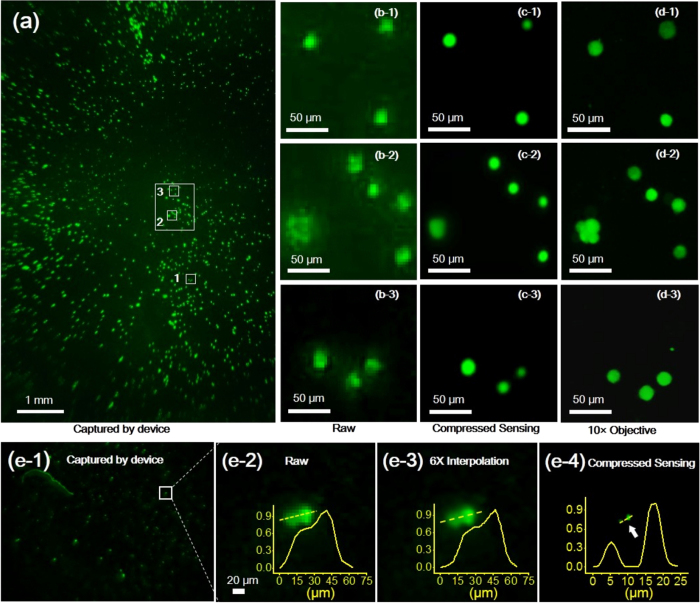
Effects of compressive sensing on improving image resolution. (**a**) The fluorescent image captured by w-SCOPE, under 0.3X magnification. (**b**) Three cropped subsections of the raw image with ~20 μm resolution. (**c**) shows the results of compressive sensing on the cropped images. (**d**) displays images of the same subsections as seen through a 10 × objective lens (N.A. 0.3) acquired via a fluorescent microscope for comparison. The cells in (**c,d**) have the same pixel spacing, indicating that through image processing of compressive sensing, we can achieve resolving power similar to a 10 × objective lens of a conventional fluorescent microscope. It should be noted that the compressive sensing algorithm is less effective in dealing with dense objects, therefore the cell cluster in Fig. (d-2) cannot be revealed up in Fig. (c-2) from the original Fig. (b-2). (**e**) shows the resolution test on sub-pixel fluorescent particles (average size ~2μm). The raw images acquired under the 0.3X magnification are presented in the left 2 columns, e-1 and e-2. A 6X bi-cubic interpolation is applied to the raw image and shown in the middle column, e-3. The right column, e-4, shows the final result by compressive sensing computation. The intensity distributions are plotted along the labelled yellow lines, for indicating the resolving power under each configuration.

With the 0.3X magnification used in the w-SCOPE imaging, we harvest a large FOV, which is very beneficial for observing dynamics of cell population, but the corresponding spatial resolution of the raw images is only approximately 20 μm^10^,^32^, which limits further analysis of single cell events such as cell/pathogen detection and quantification^8^. We imaged an MCF-7 cell line stained by CFDA SE using the w-SCOPE. The original results are displayed in Fig. 3a, in which three areas (b1–b3) are selected and cropped out from the raw fluorescent image, to show the unsatisfactory details of MCF-7 cells. This insufficient resolving power can be improved through a variety of post-processing methods. Here we used compressive sensing, which is a well-known method that can recover signals from imperfect measurement, to process the selected images shown in Fig. 3(b1–b3, [Fig f2], [Fig f3]). A 6X interpolation was applied to the raw image and an iterative interior-point least square solver^23^ was used to perform the compressive sensing computation, resulting in the recovered high resolution image with finer grid size ~0.75 um. The final enhanced results that correspond to b1 to b3 are shown in c1 to c3, respectively. Aside from one area that contains cell aggregation and forms a dense fluorescent signal, the majority of the blurred cells that are adjacent to each other in the original images can be significantly improved and clearly discerned with sharp resolvable boundaries. For direct comparison, the same zoomed regions of the cells were also imaged by a conventional fluorescent microscope (Nikon Eclipse TE-2000) mounted with 10×/0.3 objective (Nikon Plan Fluor DL 10X). The results are correspondingly shown in Fig. 3(d1–d3, [Fig f2], [Fig f3]). To verify the efficacy of the enhancement, we performed the resolution test using the fluorescent micro-particles as target (Sperotech, FH-2045-2, light yellow emission, average size 2 μm). The raw images acquired under the 0.3X magnification are presented in the left 2 columns of the Fig. e-1 and e-2, with a coarse pixel spacing of 4.5 μm and a poor resolution of ~20 μm shown by the FWHM of the resolved particles. In the middle column (e-3), the images are processed with a 6X bi-cubic interpolation which however didn’t substantially improve the resolution. The adjacent particles remain blurred together and difficult to be distinguished. After the compressive sensing being applied, the particles become much more resolvable with a significantly reduced FWHM of 3 to 4 μm (e-4, yellow plot). By combining the compressive sensing enhancement with w-SCOPE imaging, a large scale cell image containing improved resolution can be obtained for comprehensive image-based analysis, such as cell counting, mobility tracking, *etc.*, on either a few localized cells or the global large population.

### Quantitative Time-course Study of Cell Response to Paclitaxel Treatment

The *in vitro* study of cell response to drugs is an important area of drug discovery research. Various cell viability assays, such as the MTT assay and the CellTiter-Blue assay, are currently the dominant methods to quantify the cell response to a drug. Each of these assays function as end-point tests and cannot be used to assess the dynamics of drug response and cell viability in the middle of the experiment^33^,^34^,^35^,^36^. Here we propose an alternative method to conduct the time-varying cell viability analysis by using the w-SCOPE to record images of living cells exposed to a drug treatment over a period of time and then counting viable cell numbers at specific time points from the captured images. As a demonstration of this technique, we conducted a drug experiment in which paclitaxel (Taxol), a well characterized anticancer drug, was used to treat the prepared cell lines. Paclitaxel stabilizes the microtubule polymer and protects it from disassembly, preventing chromosomes from achieving a metaphase spindle configuration and blocking the progression of mitosis. Prolonged activation of the mitotic checkpoint triggers apoptosis or reversion to the G-phase of the cell cycle without cell division^37^. Paclitaxel has been widely used in cancer chemotherapy.

**Figure 4:**
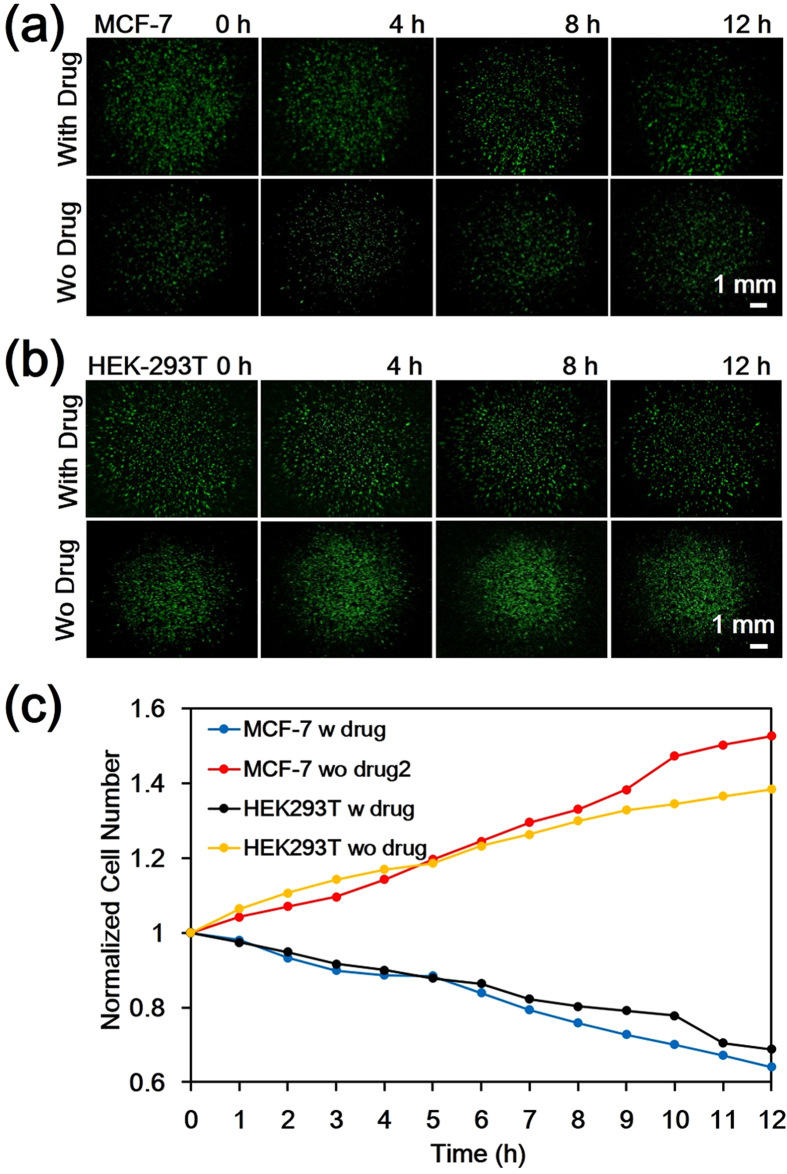
Time-course of cell response to paclitaxel anti-cancer drug treatment. (**a,b**) Representative fluorescent images for GFP-labelled MCF-7 and HEK293T cell line, respectively, with paclitaxel treatment (upper row) and without (lower row) at 0, 4, 8 and 12 hours. All the images are taken by the w-SCOPE with magnification around 0.5X. (**c**) The plot of viable cell population counted over time from 0 to 12 h at 1 h intervals for both MCF-7 and HEK293T cell lines including the paclitaxel treatment group and control group.

We used the w-SCOPE to observe the cell dynamics in response to paclitaxel treatment. Two types of cell lines, MCF-7 human breast adenocarcinoma cell line and HEK293T human embryonic kidney cell line, were prepared for target trial and control trial, respectively. Both cell lines were labelled with green fluorescence protein (GFP) for prolonged fluorescence imaging. Each cell line group was further divided into two sub-groups: a paclitaxel treatment group and a control group without adding drug. In the paclitaxel treatment group, cell culture medium (Dulbecco’s Modified Eagle’s Medium (DMEM), ATCC) with 10 μM paclitaxel was loaded into the medium petri dish at time 0 hours, while the control group was given the pure medium. All cells were incubated in a humidified 5% CO_2_ environment at 37 °C. Wide FOV fluorescent images (~0.54×) were taken at 1 hour intervals over a total duration of 12 hours. Representative time-lapse images are shown in Fig. 4(a,b). For quantitative analysis, we counted the number of viable cells in each image using an automatic nuclei counting algorithm combined with a floating cell separation method, both of which are detailed in the methods section. These results are plotted as shown in Figure 4(c). In the paclitaxel-treated groups, the numbers of viable MCF-7 tumor cells and HEK293T normal cells both decreased over time while the cell groups not exposed to the paclitaxel treatment both proliferated to a larger population. This is consistent with the prior knowledge that paclitaxel can inhibit cell proliferation and cause apoptosis. Fig. 4(c) further reveals that in the control group, MCF-7 proliferates faster than HEK293T, which accords with the truth that tumor cells usually have a faster proliferation rate than normal cells. In the paclitaxel treatment groups, the number of MCF-7 and HEK293T cells was found to be 2137 and 1881 respectively at 0 h. At the end point of 12 h, the cell count for MCF-7 decreased by 36%, while the cell count for HEK293T decreased by 30% (Fig. 4(c)).

**Figure 5:**
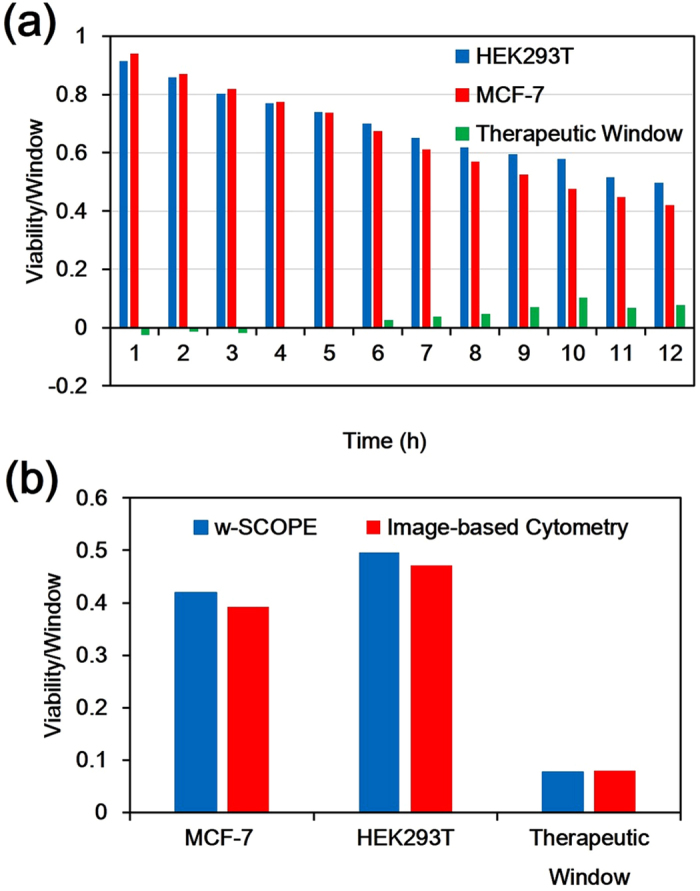
Cell viability and therapeutic window calculation based on the w-SCOPE images. (**a**) Cell viability and corresponding therapeutic window for MCF-7 and HEK293T cell lines at each hour of total 12 hour’s observation. (**b**) Comparison of the cell viability and therapeutic window results obtained by the w-SCOPE and the standard image-based cytometry method. All key experimental conditions were carefully maintained the same for strict comparison. The acceptable deviation between two sets of data verifies that the w-SCOPE is qualified to do cell viability measurement in drug screening.

After obtaining the number of cells over time, the time-varying cell viability can be calculated by using the following equation:


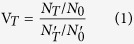


where V_*T*_ refers to the cell viability at time point T after drug administration, *N*_*T*_ and *N*_0_ are the number of cells treated with drug at time point T and at the time observation begins respectively, and 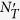 and 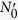 are the number of cells without drug treatment at time point T and at the time observation begins respectively. According to equation (1) and the data in Fig. 4(c), the cell viability of both cell lines and the corresponding therapeutic window (the viability of MCF-7 minus the viability of HEK293T) for each hour can be determined as shown in Fig. 5(a). By revealing the dynamic cell response to the drug rather than the common end-point test, we further found the Paclitaxel starts to take increasing positive effect from 6 h. To verify the efficacy of the w-SCOPE in assessing the cell viability and therapeutic window, we further utilized image-based cytometers (Nexcelom Bioscience LLC., Cellometer X4, USA) via bright-field analysis to perform cell concentration and viability (trypan blue exclusion) measurements as the standard method^38^,^39^ for comparison. The results are shown in Fig. 5(b). The key conditions for both methods were held constant, including drug type, drug concentration, drug exposure time and cell culturing environment. Compared to the standard cytometry method, the cell viability data obtained using the w-SCOPE for MCF-7 and HEK293T cell lines was found to have very small deviations of ~2.78% and ~2.54% respectively, which is within a standard deviation and thus deemed to be acceptable. The error between the w-SCOPE and the standard method in calculating the therapeutic window is only ~0.24%, which is negligible. Therefore, the w-SCOPE has proved to be suitable for cell viability analysis after drug treatment which is a key step in drug screening.

**Figure 6:**
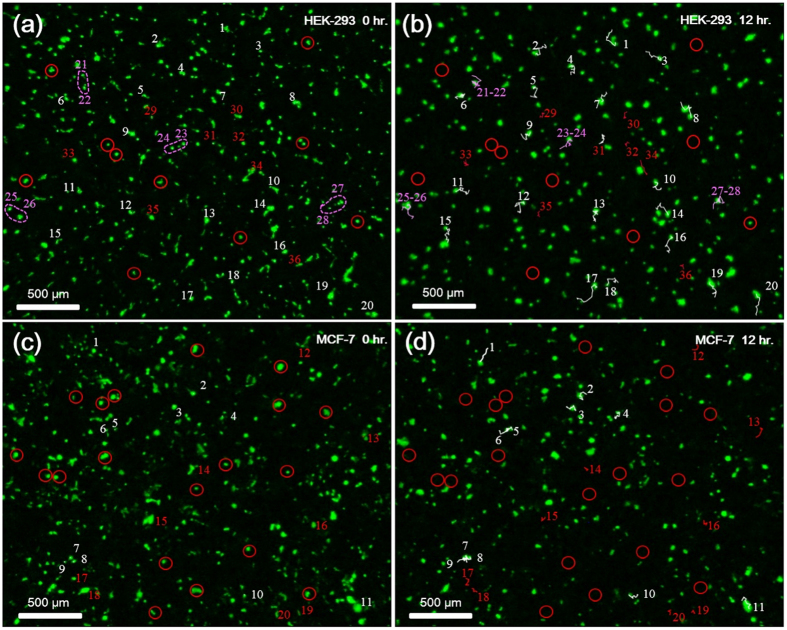
Analysis of cell motility and viability via time-lapse imaging of w-SCOPE. (**a**) The initial frame of the time-lapse images of the HEK-293 cell line, representing start time of Paclitaxel treatment, is labelled with red, purple and white points, as well as red circles indicating cells that move or disappear respectively. (**b**) The final frame of the HEK-293 trial, representing the 12 hours after Paclitaxel treatment, is shown. White colored trails indicate the paths of cells that migrated during the time-lapse period. Red trails indicate the paths of cells that migrated and eventually died before the end of the period. Purple colored trails indicate the merging of two or more cells. Red circles indicate the former location of cells that died without obvious moving. (**c**) to (**d**) The initial frame and final frame of the time-lapse data for the MCF-7 cell line treated with paclitaxel are similarly labelled and analyzed. When comparing (**b**) and (**d**), it is shown that MCF-7 cell line displays relatively higher mortality compared to motility following exposure to paclitaxel, and the HEK-293 line displayed comparatively higher motility and tendency of aggregation.

In addition, further time course study of cell response to drug, involving cell motility, aggregation and viability, can be directly visualized from the time-lapse images, as shown in Fig. 6. To perform this analysis, corresponding areas of interest were cropped out of each frame of the 12-hour HEK-293 and MCF-7 drug-exposed imaging studies and registered. Once the images in each sequence were aligned, a cell mobility analysis (MTrackJ, Biomedical Imaging Group Rotterdam, Netherlands) was applied to identify and track selected cells’ fate through each time-lapse study. In Fig. 6, white trails indicate cells that move and survive through the entire time period, red trails indicate cells that move and die prior to the end point of the study, purple trails indicate cells that merged during the time period and red circles indicate cells that died without moving. In Fig. 6a,b, we found that cells in the HEK-293 line showed, in general, a greater tendency to migrate and merge together following application of the drug as compared to the MCF-7 line. Conversely, in Fig. 6c,d, we found that a comparatively greater amount of cells died and disappeared with little movement by the final frame of the time-lapse image. These visualized quantifications consistently indicate that HEK-293 cells demonstrate greater motility, higher tendency to aggregate, and lower mortality when exposed to paclitaxel than the MCF-7 cells. The time course showing the 4 types of dynamics of the selected cells throughout all the time points of the drug exposure are further provided in the supplementary materials (Video.S1 and Video.S2).

## Discussions

The w-SCOPE is designed for continuous, on-site observation of cell dynamics occurs inside commercial incubators. Considering the frequent imaging over time and very resource-limited environment in the incubator, we use the lens-based design which is free from scanning apparatuses and multi-frame recording at one time point. At the same time, we made the device wireless so that it can be fully sealed inside the incubator and work alone via remote control and on-air data transfer using a smartphone. Considering the Petri dish has become a staple in laboratory work on both prokaryotic and eukaryotic cells over the last century^40^, the design of the device is fully compatible with standard cell culture procedures using commercial petri dishes, which ensures the *in-situ* observation of the cells with less undesired interference brought in at the same time. Furthermore, the incorporation of consumer electronics & optics significantly reduces the size and cost of the w-SCOPE. The involvement of Sony’s state-of-art digital camera that packages zoomable lens, wireless communication modules, lithium battery in a small 62 × 62 × 33 mm format, drastically simplifies the device assembling while bringing novel functionality. The use of a high-power, remotely switchable LED as the light source creates a suitable balance between having sufficient excitation light intensity for imaging, and avoiding photobleaching and phototoxicity in fluorescent imaging^41^. All components used to build the w-SCOPE are listed in Table S-1.

The ability to look into the intermediate states of a longitudinal cellular process is the highlight of the w-SCOPE. As a demonstration, we treated tumor line MCF-7 cells and control group HEK293T cells with anti-cancer drug paclitaxel and obtained the time course of cell viability, to evaluate drug efficacy with higher temporal resolution than before. To validate the viability results obtained by the w-SCOPE, we conducted currently established standard cell viability assay methods, such as image-based cytometry and CCK-8 assays, for comparison. Unfortunately, none of the current established methods are able to yield data at intermediate time points during the experiment. This precluded comparisons between data from the w-SCOPE and data from the standard cytometry and assay methods. Because of this, we compared only the results acquired at the point when drug treatment ended. The proximity between our result and image-based cytometry result strongly indicates that the w-SCOPE can be used for a cell viability assay for drug screening and also provides support for the accuracy of intermediate viability results. We also performed a CCK-8 assay and the comparison is shown in Fig. S-1. In this protocol, we set 12 h as our drug effecting time to demonstrate the system’s longitudinal study capability. In the next step, we plan to realize two days’ and even three days’ drug assay which is much more common in the field of drug discovery. Besides the drug study, the w-SCOPE can also be applied to many other types of time-lapse cellular analysis such as cell motility studies, cell proliferation studies and cell adhesion and expansion studies. We give an example of the potential of the w-SCOPE in cell motility studies in Fig. S-3.

In the process of counting cells in the fluorescent images we captured, we noted that in near end of the drug treatment process, some cells floated into the culture medium due to drug induced cell adhesion reduction. However, these cells emitted fluorescent light and were able to be imaged. In order to confirm that these cells were still alive and viable, we pipetted the culture medium out carefully at the end of drug treatment and then measured the floating cell viability using the above mentioned image-based cytometer. The average viability for the MCF-7 and the HEK293T cell lines were around 90% and 80% respectively, which indicated that the majority of floating cells are still alive. We used this viability data to create a ratio of floating live cells to total floating cells and multiplied the number of floating cells by it to obtain a more accurate viability assessment.

## Conclusion

The w-SCOPE elucidated here has a tunable field-of-view to accommodate various types of cell culture and has sufficient resolving power to conduct imaging-based cell analysis. Due to its compact form factor and wireless design, the device can be easily integrated into an incubator to conduct long-term, on-site observation on live cells via remote control from a smartphone or computer. Further, the w-SCOPE enables live cell counting and tracking, based on images captured at different time points and gives further insight into the dynamics of cell mobility, viability and the therapeutic window under sustained drug treatment. Compared with the regular end-point viability/therapeutic detection, we achieved improved temporal resolution on the viability analysis and were able to identify 6 hours as a possible turning point at which the cancer and normal cells start to show differing responses to the drug, indicating an increasing therapeutic window at this point. The quantitative results from w-SCOPE are validated by standard cell viability assays, including the CY8 assay and an image-based cytometer. Moreover, the w-SCOPE is easily implemented and highly cost effective for resource-limited environments and compatible with most commercial incubators in the market. As a result of this, besides its uses in *in vitro* drug discovery, the device also can be easily applied for a variety of biological research, such as tissue engineering, embryogenesis, stem cell differentiation & migration, in which the study of large scale dynamic cell events are in high demand.

## Materials and Methods

### Sample Preparation

#### IMR-90 and MCF-7 cell sample staining

IMR-90 (product #CCL-186) was purchased from ATCC, Inc. (USA). Vybrant^®^ CFDA SE Cell Tracer Kit (product #V12883, excitation/emission maxima: 492/517 nm) was purchased from Life technologies, Inc. (USA). To prepare labeled live IMR-90 cell samples, IMR-90 cells were grown inside a petri dish (Corning, USA) filled with the culture medium DMEM (life technologies, USA) supplemented with 10% FBS (life technologies, USA) to induce an adherent state. 2.5μL 10mM CFDA SE solution was added into 5mL DPBS (life technologies, USA) for dilution. When the cells reached the desired density, the culture medium was removed from the dish and the aforementioned DPBS with the probe was added inside. After incubating the cells for 15 minutes at 37 °C, the loading solution was replaced with fresh, pre-warmed culture medium and cells were incubated for another 30 minutes at 37 °C. Finally, the old medium was again replaced with fresh and pre-warmed medium for washing.

### MCF-7 and HEK293T cell sample GFP labeling

Both MCF-7 and HEK293T cell lines were stably transfected with EGFP fluorescent protein. Briefly, both cells were infected at approximately 35% confluence with a lentivirus carrying EGFP coding sequence driven by a CMV promoter. After 7 days cultures, cell populations with stable EGFP expression were sorted by the fluorescent activated cell sorting (FACS) with a relative consistent fluorescent expression intensity. Stably infected MCF-7 and HEK-293T cells were further propagated in DMEM supplemented with 10% FBS for expansion.

### Cellometer Image Cytometry Protocol

MCF-7 and HEK293T cell lines were both divided into two groups: 10 μM paclitaxel treatment group and none-drug group. Four groups were cultured in a 12-well culture plate with each group occupying 3 wells at the humidified 5% CO_2_ and 37 °C environment. After 12 h, the 12 cell samples were collected for each well individually after trypsin treatment and then the culture medium was replaced by 0.5 mL DPBS for each sample after centrifuging. After re-suspension, 10 μL cell suspension was pipetted and mixed with 10 μL trypan blue (Sigma-Aldrich, USA) for every sample. 12 sets of the mixture were analyzed for percent viability using the Cellometer Auto X4 instrument. For each group of cells, 3 data were averaged to get the final viability result.

### Enhanced Resolution of Remote Microscopy via Compressive Sensing

The resolving power of our remote microscope system can be improved by post acquisition digital signal processing via compressive sensing algorithms. Compressive sensing, also known as compressive decoding or compressive sampling, is a signal processing technique which aims to recover an original sparse signal based on a subsampling of measurements in which the sampling rate is below the traditional rate defined by the Nyquist-Shannon sampling theorem. Compressive sensing has been shown to be effective in improving the spatial resolution of undersampled images captured by wide-field fluorescent microscopy^8^. In our work, we employed a similar processing algorithm as a supplement to our imaging system to achieve image resolution that we would not be able to obtain with optics alone.

Mathematically, the recovery of a signal by compressive sensing can be represented by the following *l*_*1*_ least squares problem:


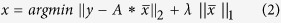


where *λ* is a regularization parameter, y is the detected raw fluorescence image, A is the two dimensional convolution matrix based on the point spread function of the system, 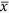 is the theoretical arrangement of fluorescent light sources that produces the image captured by the camera, and 
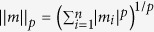
 is the *l*_*p*_ norm of m. For our processing purposes, we estimated the PSF of our system to be a two dimensional Gaussian function. The solution to this problem can be determined via an iterative interior-point solver^42^.

### Automated Cell Counting

Automated quantification of cell population from the captured fluorescent images was done through the Automatic Nuclei Counter plug-in for ImageJ (National Institutes of Health, USA). Before counting, we performed a background subtraction on each image to remove disruptive noise from our images and then adjusted the dynamic range of each image to improve the visibility of the cells against the dark background.

To prepare our images for the Automatic Nuclei Counter, we first invert the pixel values in ImageJ so that cells appear dark on a light background, and convert the image from an RGB-type to an 8-bit type. The dynamic range can be optionally adjusted here again, if necessary, to provide further contrast between the cells and the background. The Automatic Nuclei Counter accepts as inputs the image to be analyzed, a distance parameter corresponding to the estimated diameter of a cell, the approximate distance between cells and a threshold value representing the quality of cells to be detected. Cells are quantified based on the amount of dark peaks present in the image.

To identify and exclude non-viable cells from the cell counts, two images were taken at each time point. In one image of each pair, the container was gently shaken to dislodge non-adherent cells. An image subtraction was performed between the two images of each pair, resulting in a third image which displays only non-adherent cells. The number of cells in this third image was quantified and multiplied by a factor corresponding to the viability ratio of non-adherent cells, as verified by an image-based cytometer, allowing us to estimate the amount of viable cells among the floating cells. A second image subtraction is performed between the third image and the original image, resulting in an image in which only fixed cells are shown. A subtraction example based on the paclitaxel treated MCF-7 cells is provided in the Fig. S-5. Finally, the number of fixed cells is added to the number of viable floating cells for the final cell count.

## Additional Information

**How to cite this article**: Jin, D. *et al.* Compact Wireless Microscope for *In-Situ* Time Course Study of Large Scale Cell Dynamics within an Incubator. *Sci. Rep.* **5**, 18483; doi: 10.1038/srep18483 (2015).

## Supplementary Material

Supplementary Information

Supplementary Video S1

Supplementary Video S2

Supplementary Video S3
