# Supplementary material for: Compact Wireless Microscope for In-Situ Time Course Study of Large Scale Cell Dynamics within an Incubator
Source: Sci Rep. 2015 Dec 18;5:18483. doi: 10.1038/srep18483 (PMC4683435; doi:10.1038/srep18483)
Supplement: Supplementary Information [file srep18483-s1.pdf]

Supplementary Information

Compact Wireless Microscope for In-Situ Time Course Study of Large Scale Cell Dynamics within an Incubator

Di Jin,[2] Dennis Wong,[4] Junxiang Li,[5] Zhang Luo,[6] Yiran Guo,[7] Bifeng Liu[7], Qiong Wu,*[5] Chih-Ming Ho*[3] and Peng Fei*[1,3]

[1] School of Optical and Electronic Information, Huazhong University of Science and Technology, Wuhan, 430074, China.

[2] Department of Precision Instrument, Tsinghua University, Beijing, 100084, China.

[3] Mechanical and Aerospace Engineering Department, University of California, Los Angeles, Los Angeles, 90095, U.S.A.

[4] Department of Bioengineering, University of California, Los Angeles, Los Angeles, 90095, U.S.A.

[5] School of Life Sciences, Tsinghua University, Beijing, 100084, China.

[6] School of Mechanical and Engineering, Huazhong University of Science and Technology, Wuhan, 430074, China

[7] College of Life Science and Technology, Huazhong University of Science and Technology, Wuhan, 430074, China

Supplementary materials:

**Tab. S-1**	Parts information of w-SCOPE, including specification, cost, manufacturing method and vendors
**Fig. S-1**	The pictures of the w-SCOPE parts and the whole device working in an incubator
**Fig. S-2**	Comparison of the cell viability and therapeutic window results obtained by the w-SCOPE and the standard CCK-8 assay
**Fig. S-3**	Demonstration of w-SCOPE's capability of conducting cell migration study
**Fig. S-4**	A 72 hours' time-course study on HeLa cervix cell culture using w-SCOPE
**Fig. S-5**	The image subtraction for separating the floating and non-floating cells

**Video. S1**	Video showing the comprehensive dynamics of the selected HEK-293 cells throughout all the time points of the Paclitaxel exposure
**Video. S2**	Video showing the comprehensive dynamics of the selected MCF-7 cells throughout all the time points of the Paclitaxel exposure
**Video. S3**	Video showing that dividing cells transiently turn round but still attach to the dish surface

All the parts used for the w-SCOPE are compact and cost-effective. The complete part information, including function, specification, price and vendor, are listed in Table. S-1. It should be noted that the cost of 3d printing could drastically drop by either batch production or changing the vendor.

Part name	Number	Unit price (U.S. dollar)	Note	Vender	Total (U.S. dollar)
Front Cap Mover	1	345	3D printing	Partsnap	345
Front Cap Base	1				
Petri Dish Holder	1				
Main body	1				
Back cap	1				
Achromatic Doublet Lens	1	85	f=30 mm, d=25.4 mm	Thorlabs	85
Fluorescence Imaging Filters(GFP)	1	65	Ø25 mm		65
Lens holder	1	10	For doublet lens		10
Digital Camera	1	198	f=4.45-44.5 mm	Sony	198
Soft tip screw	10	0.5	M4×0.7, Length=6 mm	McMaster-CARR	5
Clamping screw	3	1	M9.4×1, Length=20 mm		3
Battery	1	7	CR2477 with holder	Dig key	7
LED	1	2	Blue, center wavelength 470 nm		2
RF Remote control module	1	11	315MHz	Amazon	11
Total					731

Table. S-1. This table shows the information of the parts that are used on w-SCOPE, including specification, price, manufacturing method and vendors.

Besides the 3-D model in Fig. 1, to further display the w-SCOPE and its working principle, the real product is imaged and shown in Fig. S-1.

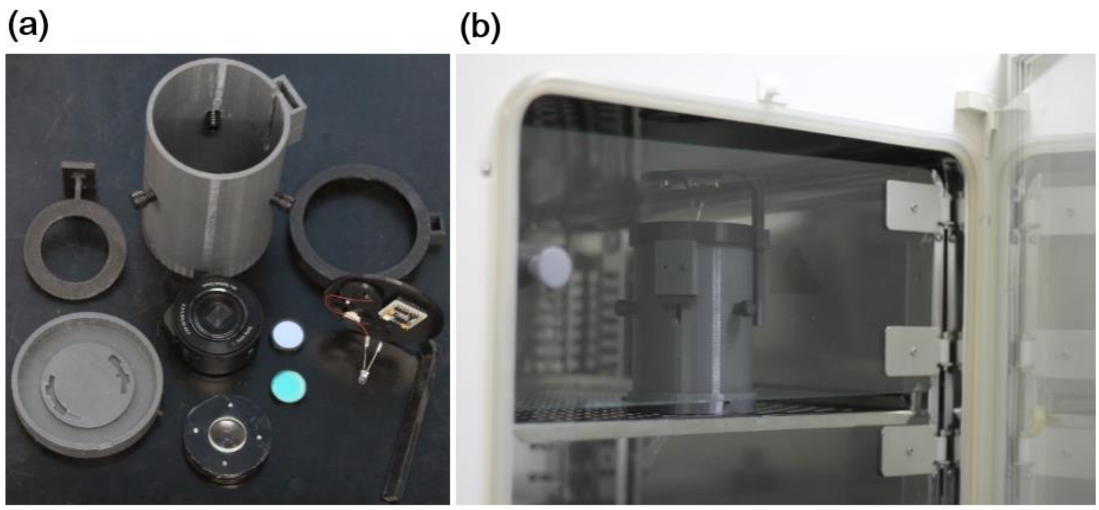

Fig. S-1. (a). The separate parts of w-SCOPE. (b). w-SCOPE working in the incubator.

The cell viability and therapeutic window results obtained from w-SCOPE have been proven to be highly consistent with those from standard image-based cytometers (figure. 5b). Here we further validate w-SCOPE's performance via comparing it with CCK-8 cell viability assay. The results are shown in Fig. S-2. For both cell lines, the cell viability results from CCK-8 assay are larger than those from w-SCOPE. A possible reason is that, though due to bad physiological conditions, some cells emit very weak fluorescent light that can hardly be detected by the w-SCOPE, they are still alive and thus can be discerned by the CCK-8 method. However despite the deviation between two methods for the cell viability measurement, their therapeutic window results remain very close, indicating a reasonable analysis from w-SCOPE.

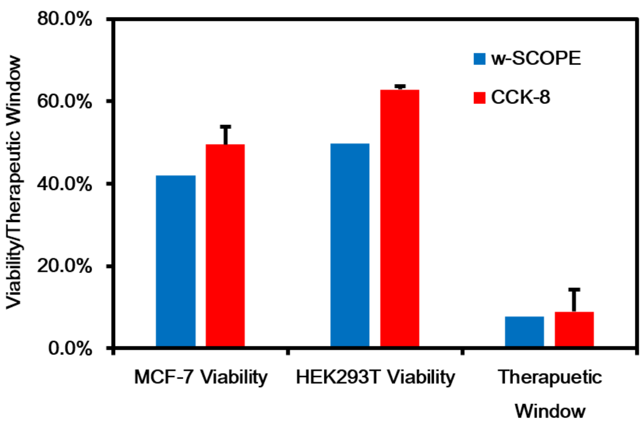

Fig. S-2. Comparison of the cell viability and therapeutic window results obtained by the w-SCOPE and the standard CCK-8 assay. All the key experimental conditions including the drug type, drug concentration, drug exposure time and the cell culturing environment had been carefully maintained the same for strict comparison and the viabilities were calculated after the cells being treated with paclitaxel for 12 h. The CCK-8 assay had been repeated for three times to generate the error bar.

Through continuous monitoring on the HEK-293T cells treated with Paclitaxel, we found when these cells were experiencing tough environment caused by the drug, they tended to migrate closer with each other (e.g., cell group C in the figure S-3) or even touched with each other and eventually merged together (e.g., cell group A and B in the figure S-3). A possible explanation of this phenomena is that the cells are trying to aggregate for a better survival from cytotoxicity. Here we gave an example that the w-SCOPE can be used to visualize the dynamics of cell migration. In the future, we plan to further perform comprehensive study on tumor cells fate, such as the study of proliferation, mobility and viability, under various anti-cancer drugs treatment.

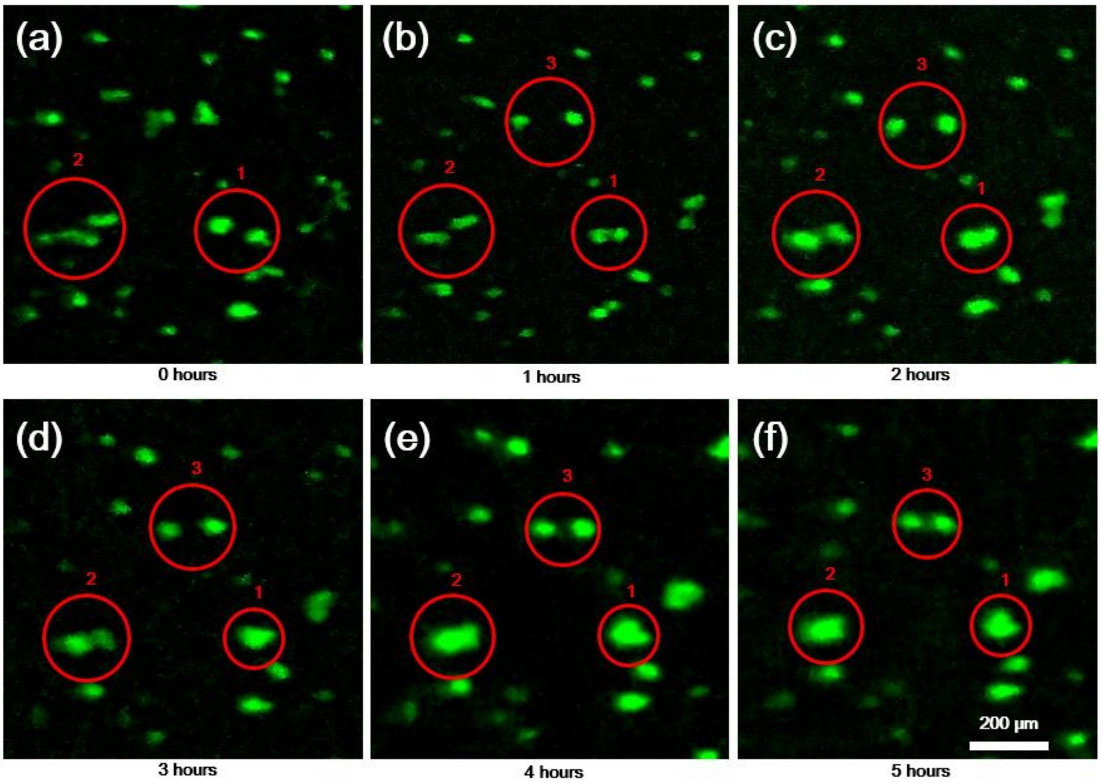

Fig. S-3. Visualization of cell migration using w-SCOPE with fluorescence configuration. The selected HEK-293T cells were incubated overnight before treated with 10 nM Paclitaxel. (a)-(f) show the time-lapse images which were captured from 1 hour to 6 hour post paclitaxel treatment, with an interval of 1 hour.

For verifying the reliability of our device, we additionally performed a long term, 72 hours cell culture on w-SCOPE using HeLa cervix cell line and continuously studied the dynamics of the cell proliferation from the start of adherence to the maximum confluence. The results are presented in Fig. S-4. The (a) to (n) shows the cell images that were recorded from start point to 72 h, with an interval of 4 hours (24 hours between the last 2 time points). Then we compare 2 small region-of-interests from 12 h and 32 h (fig. S-4, o), to further uncover the details of the cell proliferation (3 areas labelled as red). In (p), the number of the cells under each time point was counted based on the image, and its variation was further plotted in respect to the entire culture time. The plot indicates that the HeLa cells may have a quasi-linear proliferation rate before it reaches a high confluence at ~40 hours.

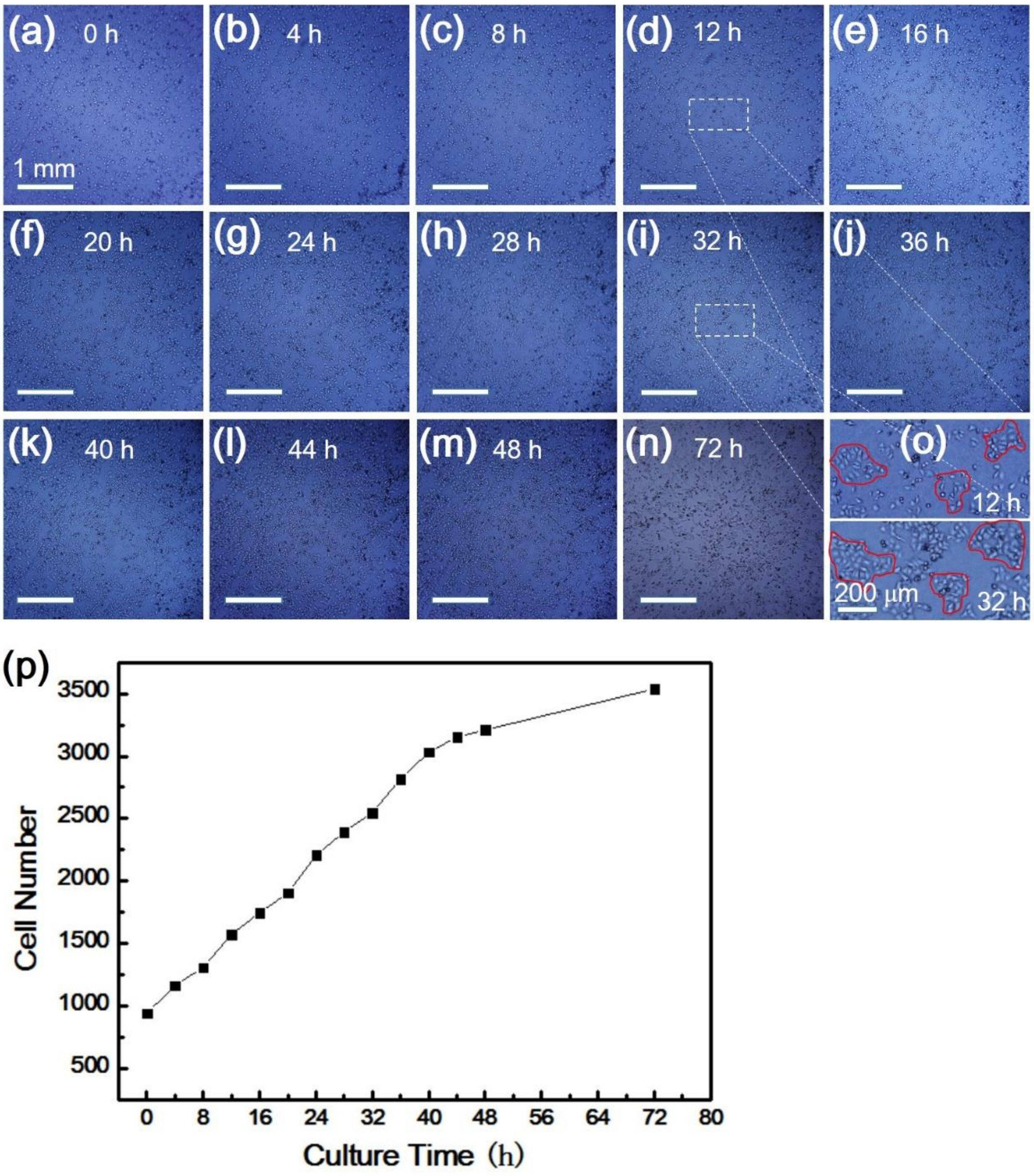

Fig. S-4. A 72 hours' time-course study on HeLa cervix cell culture using w-SCOPE. (a)-(n) show the time-lapsed cell images which were recorded from 0 hour to 72 hours. The time interval is 4 hour from (a) to (m), and 24 hour between (m) and (n). (o) compares 2 selected regions from 12 h and 32 h. The areas shows significant cell proliferation are labeled as red. (p) The plot of the cell number in respect to the culture time.

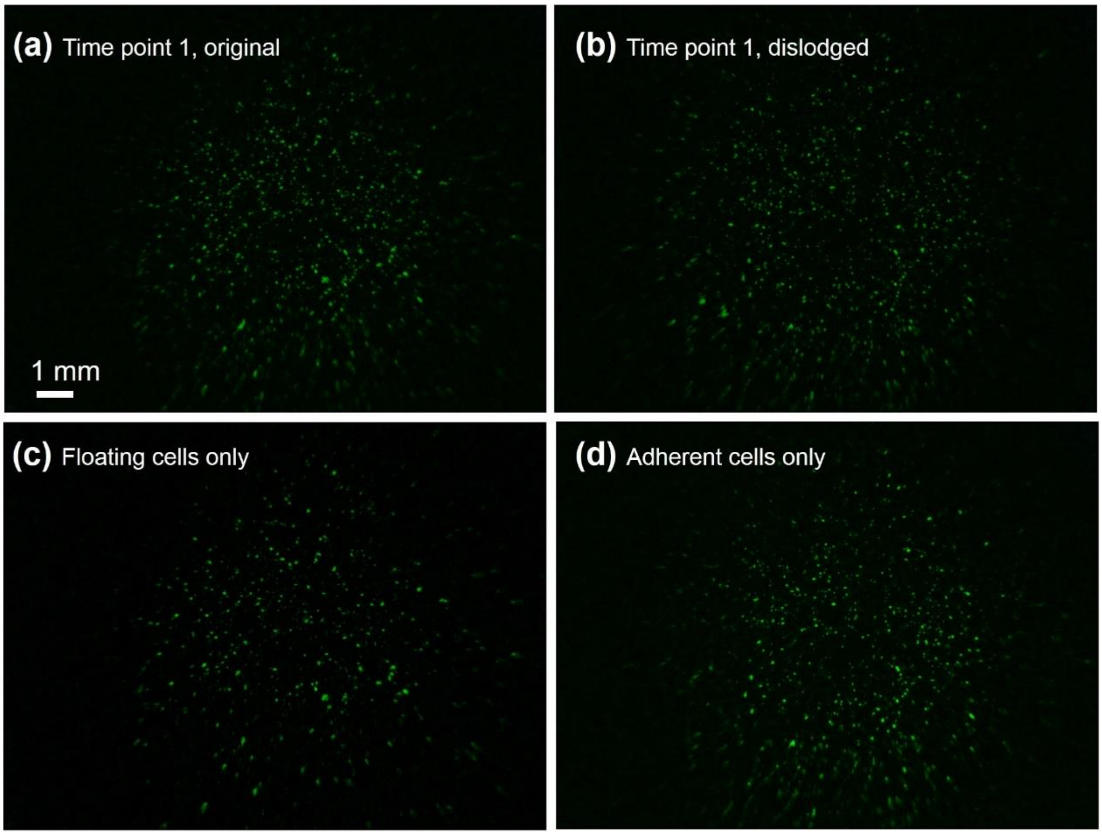

Fig. S-5. The image subtraction for separating the floating and non-floating cells. (a) The original cell image containing the floating and adherent cells. (b) The second cell image taken at the same point but with the floating cells dislodged. (c) The subtraction of the first 2 images, which generates an intermediate image displaying the floating cells only. (d) The Subtraction between the original image and the intermediate image, showing the adherent cells only.

Video. S1 shows the 12 hours time-lapse mobility tracking of the HEK-293 cells with 1 hour interval per frame.

Video. S2 shows the 12 hours time-lapse mobility tracking of the MCF-7 cells with 1 hour interval per frame.

Video.S3 shows that dividing cells transiently turn round but still attach to the dish surface.
