# Supplementary figures and images for: Compact Wireless Microscope for In-Situ Time Course Study of Large Scale Cell Dynamics within an Incubator
Source: Sci Rep. 2015 Dec 18;5:18483. doi: 10.1038/srep18483 (PMC4683435; doi:10.1038/srep18483)

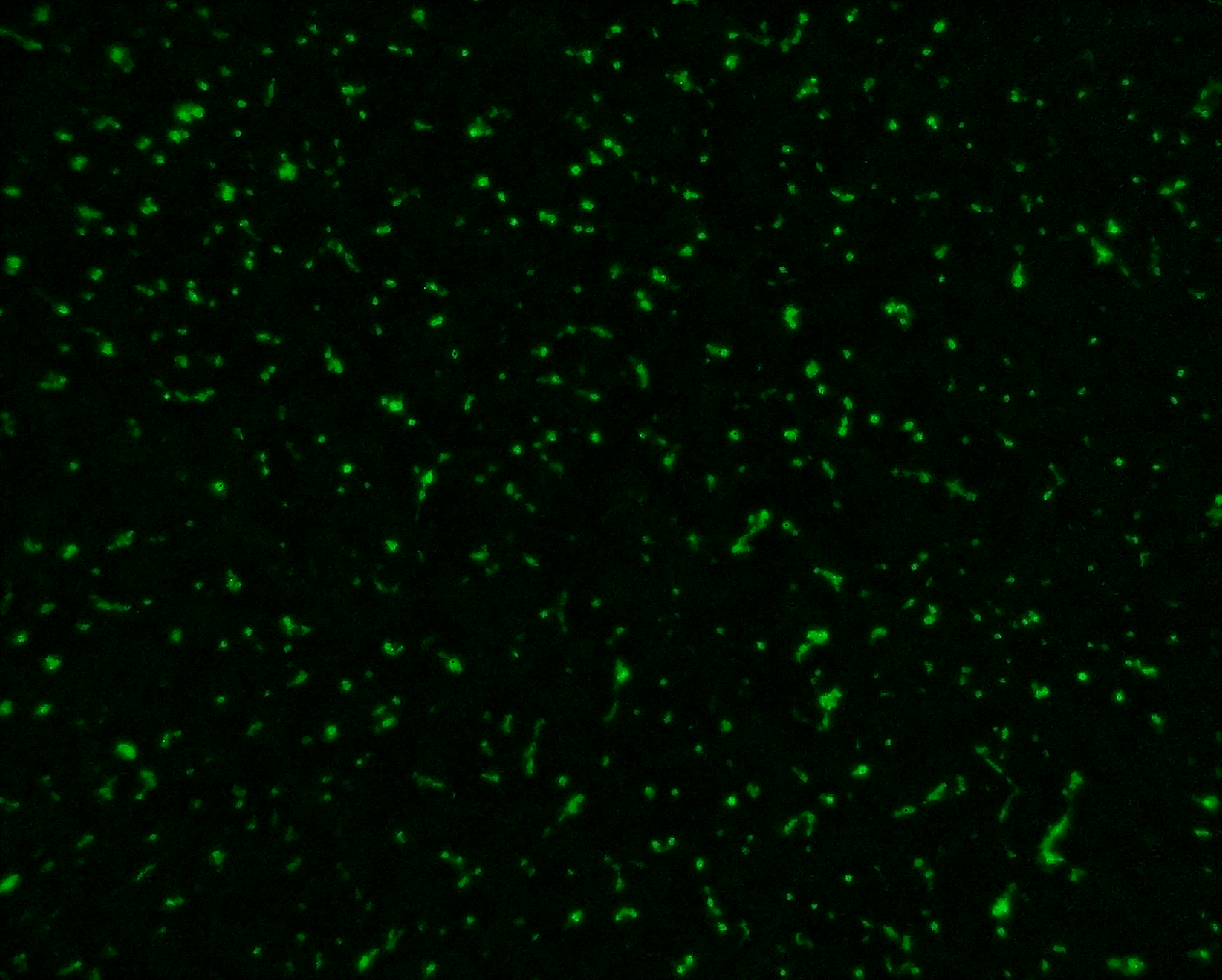

Supplement: Supplementary Video S1 [file srep18483-s2.gif]

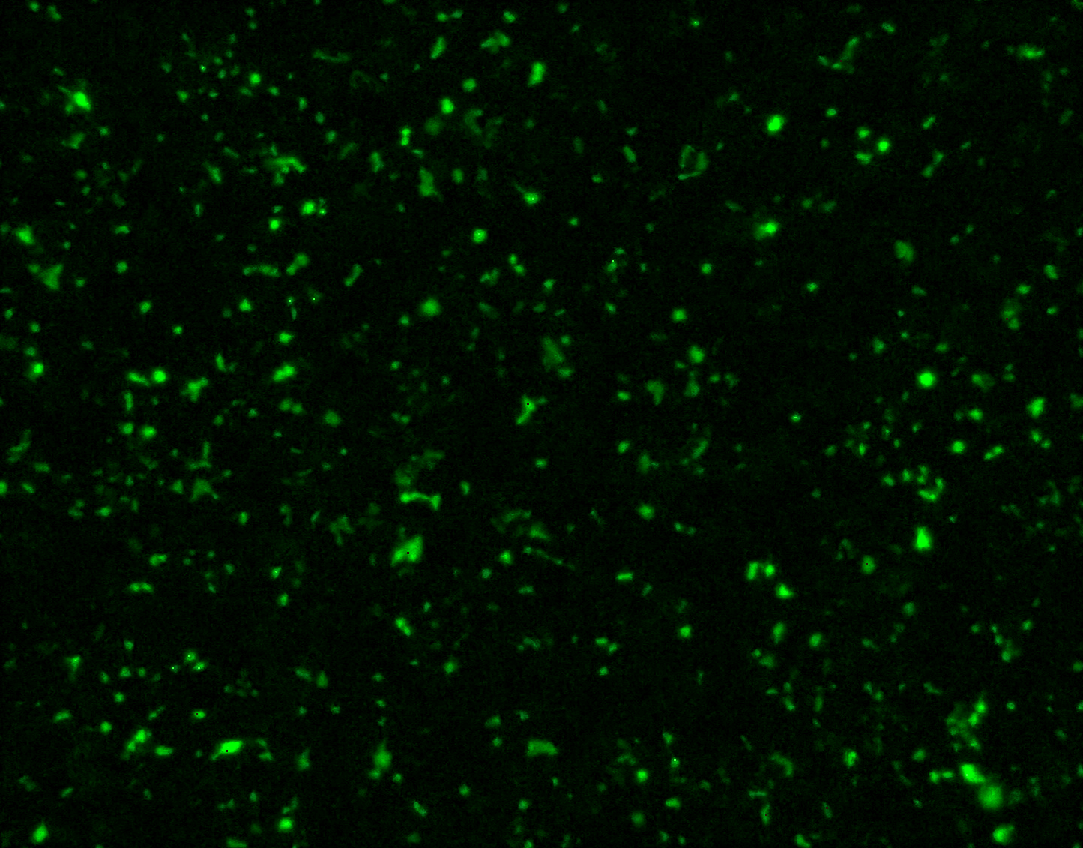

Supplement: Supplementary Video S2 [file srep18483-s3.gif]
